# Metaverse? No, thanks! Exploring the mechanisms behind Generation Z’s resistance behavior

**DOI:** 10.3389/fpsyg.2025.1672330

**Published:** 2025-11-03

**Authors:** Ning Ding, Liling Hu, Qin Zhao, Kyung-Tae Kim, Maowei Chen

**Affiliations:** ^1^Department of Global Convergence, Kangwon National University, Chuncheon, Republic of Korea; ^2^Department of Business Administration, Kangwon National University, Chuncheon, Republic of Korea

**Keywords:** metaverse, Generation Z, resistance behavior, grounded theory, SEM, fsQCA

## Abstract

The metaverse is progressively advancing toward broad application in real-world scenarios. However, as a key driving force of today’s digital economy, Generation Z has not demonstrated sufficient enthusiasm for participation. This study adopts a mixed-methods approach to systematically explore the resistance behaviors of Generation Z toward the metaverse and their underlying causes. In the first phase, grounded theory was employed to analyze data from 25 in-depth interviews. Through three levels of coding, seven key resistance factors were identified: interpersonal alienation, psychological burden, social norm conflict, value doubt, perceived complexity, perceived unavailability, and perceived risk. In the second phase, structural equation modeling (SEM) was used to examine the net effects of these factors on resistance behavior. The results indicate that all factors except perceived complexity have a significant positive influence on resistance behavior. In the third phase, fuzzy-set qualitative comparative analysis (fsQCA) was employed to identify nine configurations of conditions that lead to resistance, thus addressing the limitations of SEM in capturing complex causal relationships. This study not only extends the theoretical boundaries of user behavior research in the metaverse context but also provides empirical insights for platforms aiming to optimize user experience and develop operational strategies targeted at Generation Z.

## 1 Introduction

With the accelerated convergence of generative artificial intelligence, immersive technologies, and blockchain systems, the metaverse is gradually evolving from a futuristic technological vision into a digitally hybrid space where the virtual and real worlds are deeply intertwined (Nguyen et al., 2023). Its applications have permeated various sectors—including education, entertainment, social interaction, and healthcare—demonstrating considerable potential for cross-sector integration and transformative innovation (Wang Y. et al., 2025). Since Facebook rebranded as Meta in 2021 and launched a series of virtual interaction products, the metaverse has rapidly emerged as a strategic priority among global technology companies, attracting significant attention and sustained investment from capital markets (Qadir and Fatah, 2023; Kaplan and Haenlein, 2024). Although a fully functional and immersive metaverse remains a forward-looking aspiration (Mvondo, 2025), a number of augmented and virtual reality platforms—such as The Sandbox, Zepeto, Roblox, Horizon Worlds, Gather Town, and Decentraland—have already been launched, offering an initial glimpse into the contours of a digital future (Wu and Yu, 2024). According to the latest report from Fortune Business Insights (2025), the global metaverse market reached a size of USD 737.73 billion in 2024. It is projected to grow from USD 1,273.58 billion in 2025 to USD 7,639.7 billion by 2032, with a compound annual growth rate of 29.2% over the forecast period—underscoring strong industrial expansion potential and promising economic prospects. However, despite the high level of synergy between technological advancement and capital investment, user responses at the behavioral level have proven to be complex and heterogeneous (Xing and Zhang, 2025). As of 2024, the global number of metaverse users exceeded 600 million, with 80% of active users aged 16 and below (Market.us, 2024). Notably, despite being an increasingly important demographic in today’s digital economy (Yani and Santosa, 2024), Generation Z has not demonstrated sufficient enthusiasm for participation (Korn et al., 2024; Kaabachi et al., 2025). Against this backdrop, it is essential to adopt a multidimensional perspective to examine Generation Z’s cognitive evaluations, emotional experiences, and social motivations during the stages of perception and anticipation, thereby systematically uncovering the underlying indifference and potential mechanisms of psychological disengagement that characterize their attitudes toward the metaverse.

Currently, scholarly research on the metaverse is extensive, encompassing multiple dimensions such as technological architecture (Wang Y. et al., 2025), industrial ecosystems (Qadir and Fatah, 2023), ethical governance (Al-Kfairy et al., 2025a), and legal regulation (Qin et al., 2025). Among these, studies on user behavior have predominantly focused on facilitating factors, emphasizing the positive influences of perceived usefulness, immersive experiences, and social interaction on users’ adoption intentions (Nguyen et al., 2023; Wu and Yu, 2024). However, research on user resistance and discontinuance behavior remains relatively limited, with a particularly notable scarcity of systematic investigations targeting Generation Z users. This research gap further underscores the theoretical importance and practical relevance of the present study. In addition, existing studies on resistance behavior are mostly built upon predetermined variable frameworks (Mvondo, 2025; Sowmya et al., 2024), with limited efforts to identify key factors emerging from users’ actual experiences and subjective perceptions. This limits the capacity to holistically capture the cognitive processing and emotional responses occurring within complex human-computer interaction scenarios. Methodologically, previous studies have predominantly employed symmetrical analytical techniques such as SEM (Wu and Yu, 2024; Gupta et al., 2024), which focus on the net effects of individual variables, yet often overlook potential asymmetric interactions and multiple concurrent causal pathways among variables. To address these theoretical and methodological limitations, the present study adopts a three-stage mixed-method research design that integrates grounded theory’s three-level coding, SEM, and fsQCA. The aim is to provide deeper theoretical insights into the underlying drivers of Generation Z’s resistance to metaverse adoption during the anticipation stage. The study primarily focuses on the following core research questions:

RQ1. What are the key factors underlying Generation Z’s resistance toward metaverse platforms, and how do these factors influence their resistance behaviors?

RQ2. Under conditions of complex interrelationships, what distinct combinations of resistance factors constitute significant configurational pathways that lead to resistance among Generation Z?

RQ3. What similarities and differences emerge between the findings of SEM and fsQCA in explaining Generation Z’s resistance behaviors?

To systematically address the aforementioned research questions, this study conducted semi-structured in-depth interviews with 25 Generation Z internet users from mainland China. The participants possessed only a preliminary understanding of the metaverse but had no actual usage experience. Data were collected through semi-structured, in-depth interviews, and analyzed using the three-level coding procedure of grounded theory to extract the key perceptual factors underlying their resistance toward metaverse adoption. Based on these findings, a theoretical model was developed by integrating the core variables. Subsequently, drawing on 392 valid survey responses, SEM and fsQCA were employed to examine and compare the path relationships and configurational mechanisms, respectively, thereby exploring the similarities and differences in explanatory logic between the two methods. This research not only extends the theoretical boundaries of user behavior studies in the metaverse domain but also provides empirical evidence to support platform providers in formulating personalized operational strategies and user retention initiatives.

## 2 Literature review

### 2.1 Generation Z

Campbell et al. (2015) defined a “generation” as “a cohort of individuals born during the same historical period and sharing a similar cultural context,” emphasizing that shared historical and cultural experiences play a significant role in shaping the behaviors and attitudes of generational cohorts. In the extant literature, commonly referenced generational groups include: the Baby Boomers (born between 1946 and 1964), Generation X (born between 1965 and 1979), Generation Y or Millennials (born between 1980 and 1994), and Generation Z (born between 1995 and 2009) (Maloni et al., 2019; Herring, 2019). Although there is no universally agreed-upon definition of the precise time span of Generation Z, most scholars generally characterize this cohort as individuals born from the mid- to late-1990s and who came of age in the early 2000s (Magano et al., 2020; Nedelko et al., 2022). Compared with preceding cohorts, Generation Z has been exposed to the Internet and smart devices from an early age, demonstrating a high degree of technological proficiency and dependence (Chan and Lee, 2023). One of the defining traits of this generation is their rapid acceptance and adaptation to emerging technologies (Wang and Zhang, 2023). Furthermore, Generation Z places significant emphasis on personal efficiency and independence (Magano et al., 2020). They rely heavily on the internet for social interaction, shopping, and entertainment, tend to acquire information through online platforms, and actively engage in content sharing (Erwin et al., 2023). This dependence on digital technology contributes to their heightened expectations for personalized and efficiency-enhancing technological products and services (Chan and Lee, 2023).

In recent years, as the technological environment continues to evolve and application scenarios become increasingly diverse, Generation Z has shown both enthusiasm for emerging technologies and a growing tendency toward resistance and avoidance (Christian et al., 2023). Existing studies suggest that concerns over privacy and security, perceived complexity of use, and the psychological burden induced by technology may constitute key factors contributing to Generation Z’s hesitancy or even resistance toward certain emerging technologies (Kaabachi et al., 2025; Mvondo, 2025). In the process of perceiving and anticipating the metaverse, Generation Z exhibits this negative attitude (Boccalini et al., 2024a), as evidenced by their relatively low engagement levels (Market.us, 2024). While prior research has predominantly focused on users’ motivations and adoption intentions (Al-Adwan and Al-Debei, 2024; Calderón-Fajardo et al., 2025), systematic exploration of the complex behavioral mechanism of psychological resistance exhibited by Generation Z in metaverse environments remains insufficient.

### 2.2 The metaverse and resistance behavior

The concept of the “metaverse” was first introduced in Neal Stephenson’s 1992 novel Snow Crash, where it was portrayed as a virtual reality space in which users engage through avatars and intelligent agents, integrating the internet with augmented reality technologies (Joshua, 2017). In recent years, this concept has been further clarified and refined. The metaverse is now widely conceptualized as a persistent, shared virtual environment constructed through advanced technologies such as VR, AR, AI, and blockchain. This environment not only simulates but also extends the functions of the real world (Al-Kfairy et al., 2025a). Among these technologies, VR and AR provide the foundational infrastructure for immersive user experiences, while AI plays a crucial role in behavior prediction and personalized content recommendations (Di Natale et al., 2024). Meanwhile, blockchain technology ensures trust and value circulation within the metaverse through applications such as smart contracts, digital asset authentication, and secure transactions (Calderón-Fajardo et al., 2025). In terms of access modalities, users can enter the metaverse via a variety of terminal devices, including traditional personal computers, tablets, smartphones, and wearable devices (Di Natale et al., 2024). Çelik and Ayaz (2025) identified three core characteristics of the metaverse: presence, interoperability, and standardization. Presence refers to the sense of immersion and co-presence that users experience within virtual environments; interoperability denotes the ability of users to transfer identities and digital assets across platforms and domains; and standardization provides the technical foundation for seamless integration and service interconnectivity among platforms. Today, the metaverse has seen widespread integration across diverse domains such as education and training (Al-Adwan and Al-Debei, 2024), cultural tourism (Calderón-Fajardo et al., 2025), and financial services (Nguyen et al., 2023), driving a paradigmatic transformation within the digital ecosystem (Wongkitrungrueng and Suprawan, 2024).

In recent years, the actual adoption of the metaverse by users has not been as optimistic as initially anticipated (Frank and Rudolf, 2024; Mvondo, 2025). A growing body of preliminary research has begun to examine the mechanisms underlying user resistance toward the metaverse. For instance, Sowmya et al. (2024), Gupta et al. (2024), and Agrawal and Wankhede (2025) have explored resistance behaviors in context-specific domains such as tourism, services, and manufacturing, respectively. However, these studies primarily focus on sector-specific applications and broad user groups, resulting in relatively singular analytical perspectives. More recently, two studies have attempted to explore resistance mechanisms from a more macro-level perspective. Drawing on Innovation Resistance Theory (IRT), Mvondo (2025) employed SEM and fsQCA to investigate psychological and functional barriers to metaverse adoption. The study identified fear of cybersickness, fear of addiction, lack of physical tangibility, and fear of identity theft as the main deterrents to usage. Similarly, Kaabachi et al. (2025), also grounded in IRT and combining grounded theory with SEM, found that perceived corporate irresponsibility, skeptical attitudes, privacy and security risks, and insufficient informational awareness were the primary barriers hindering Gen Z users in France from adopting the metaverse. Nevertheless, Mvondo (2025)‘study relied predominantly on theoretical deduction from existing literature to identify independent variables, without delving into the emerging concepts and potential factors in users’ real perceptual contexts. Although Kaabachi et al. (2025) addressed this gap through qualitative methods, the study did not further examine the configurational effects among multiple resistance factors, and its findings were limited by geographical sampling constraints. It is worth noting that both studies employ IRT, proposed by Ram and Sheth (1989), as their theoretical framework. As a classic theory of user resistance behavior (Ma and Lee, 2019), IRT highlights the explanatory power of functional barriers (usage, value, and risk barriers) and psychological barriers (tradition and image barriers) in user resistance, and has been widely applied in fields such as educational technology (Ma and Lee, 2019), digital marketing (Yang and Kwon, 2024), and mobile payment (Hameed et al., 2025). In the metaverse context, although the studies of Mvondo (2025) and Kaabachi et al. (2025) extend the IRT framework to some extent, they remain primarily focused on functional and psychological dimensions, lacking in-depth exploration of the complex emotional responses and behavioral patterns manifested in users’ perceptions and anticipations prior to metaverse adoption. This shortcoming not only constrains a systematic understanding of user resistance mechanisms in the metaverse but also weakens the effectiveness of practical intervention strategies. Hence, there is an urgent need to adopt more open-ended exploratory approaches to identify novel barriers in users’ authentic contexts, thereby enriching and extending the explanatory capacity of IRT and offering more targeted practical insights for the sustainable development of the metaverse.

## 3 Extraction of resistance factors among Generation Z

### 3.1 Data collection

As a classical qualitative research methodology, grounded theory is widely regarded as particularly suitable for investigating emerging fields that remain underexplored or lack well-established theoretical frameworks. It enables the systematic derivation and construction of new theoretical models directly from raw empirical data (Ahmed and Haag, 2016). The three-tiered coding process advocated by grounded theory—comprising open coding, axial coding, and selective coding—has been validated across a wide range of research domains. This approach is especially effective in analyzing unstructured data sources, such as interview transcripts and focus group discussions (Yue et al., 2024). In recent years, with the diversification of research paradigms, the three-level coding methodology of grounded theory has also demonstrated significant value during the early stages of quantitative research. In particular, it has proven useful for identifying latent variables and developing measurement constructs (Hu et al., 2025; Kaabachi et al., 2025).

The target population for this phase of the study comprised individuals from mainland China aged 18 to 30, representing the Generation Z cohort. Participants were required to possess a basic understanding of the metaverse concept but to have had no prior experience using metaverse platforms. To obtain authentic and in-depth first-hand data, the research team conducted semi-structured interviews and recruited participants through Xiaohongshu, one of China’s leading social media platforms. The choice of Xiaohongshu as the recruitment channel was based on two primary considerations. First, the platform centers around lifestyle sharing and consumer experience exchange among young users, and has evolved into one of the largest and most active community e-commerce and content-sharing platforms in China, with over 200 million daily active users (Peng et al., 2025). This ensures a high degree of sample representativeness and enhances the external validity of the study. Second, the platform’s comprehensive tagging system and efficient community management mechanisms provided the research team with the means to accurately identify the target population and facilitate subsequent communication (Ning, 2024), thereby significantly improving recruitment efficiency and the overall feasibility of the study. To avoid the risk of respondents misreporting their eligibility due to a vague understanding of the metaverse concept, this study incorporated an online confirmation stage after the initial recruitment. The research team distributed electronic materials that elaborated on the definition of the metaverse and introduced several widely recognized prototype platforms, including social (Second Life), gaming (Roblox), educational (Sinespace), commercial (VIVERSE), and creator economy (Cryptovoxels), together with their official websites. These resources helped respondents further verify whether they met the criterion of “never having used a metaverse platform.” The research team ultimately succeeded in recruiting 25 eligible participants (Table 1). The interviews focused on several key themes, including participants’ cognitive understanding of the metaverse, emotional responses, potential concerns, and specific factors influencing their willingness to adopt metaverse technologies. The interview phase of this study was granted ethical exemption by the Department of Global Convergence at Kangwon National University. With informed consent obtained from all participants, the entire interview process was audio-recorded. The recordings were then transcribed verbatim by members of the research team, resulting in approximately 100,000 words of high-quality qualitative data. It is important to note that, given the participants had no actual usage experience and that a fully realized metaverse has not yet materialized (Mvondo, 2025), the interview content primarily reflects Gen Z’s perceptions and anticipations of the metaverse in its early-stage forms rather than insights derived from real usage experiences.

**Table 1:** Demographic characteristics of respondents.

Items	Categories	Frequency
Gender	Male	13
	Female	12
Level of education	High school or lower	2
	Associate degree	7
	Bachelor’ s degree	8
	Master’ s degree or higher	8
Occupation type	Employed	12
	Freelancer	6
	Unemployed	2
	Student	5
Interview duration (minutes)	20–25	11
	26–30	14

### 3.2 Data coding and analysis

Upon completion of data collection, the research team divided the full set of textual materials into three parts: two-thirds were used for coding and analysis, while the remaining one-third was reserved for theoretical saturation testing. NVivo 11 software was employed to systematically manage and code the interview data. Following the procedural grounded theory approach proposed by Strauss and Corbin (1990), the team adopted a three-stage coding strategy to conduct an in-depth analysis of the data. Specifically, during the open coding phase, the researchers carefully analyzed the original interview transcripts line by line and paragraph by paragraph, guided by the study’s central themes and objectives. This process led to the identification of 41 initial concepts that reflected participants’ perceived characteristics. These concepts were then refined by consolidating semantically similar or thematically related items, while excluding those with a frequency of fewer than two mentions or those showing internal inconsistencies. Ultimately, 18 subcategories were identified. In the axial coding phase, the 18 subcategories were further clustered and revised in multiple iterations around the core research questions, resulting in the emergence of 7 higher-order main categories. Finally, the same three-stage coding process was applied to the one-third of interview data reserved for theoretical saturation testing. The aim was to verify and validate the existing conceptual and categorical framework. The analysis revealed no new categories or concepts, indicating that the conceptual model developed in this study was sufficiently complete and structurally stable, thus meeting the requirements for theoretical saturation. The detailed coding results are presented in Table 2.

**Table 2:** Coding results.

Main categories	Subcategory	Subcategory definitions
A1 Interpersonal alienation	a1 Sense of virtual alienation	Belief that virtual environments lack the warmth of real interpersonal interactions.
	a2 Feeling of social isolation	Concern that using the metaverse may weaken real-life social circles and lead to loneliness.
A2 Psychological burden	a3 Concern about addiction	Worry that using the metaverse could lead to addiction to the virtual world, affecting real-life study or work.
	a4 Discomfort with immersion	Concern that using VR devices may cause discomfort such as dizziness or nausea.
	a5 Confusion between virtual and real	Fear that prolonged use may blur the line between virtual and real identity.
A3 Social norm conflict	a6 Negative word of mouth	Media or public opinion frequently highlight risks and problems, negatively influencing personal attitudes.
	a7 Low peer adoption rate	Few friends around use or understand the metaverse, leading to a lack of shared social atmosphere.
A4 Value doubt	a8 Lack of real-world relevance	Doubt about the relevance of the metaverse to real-life goals; perceived as detached from practical needs and unable to solve real-world problems.
	a9 Uncertainty about the future	Skepticism about whether the metaverse is just a short-term trend, with unclear long-term value.
	a10 Cultural stereotypes	Belief that the metaverse lacks diverse perspectives and oversimplifies or distorts culture, raising concerns about cultural values.
A5 Perceived complexity	a11 Difficulty in understanding functions	Perception that the metaverse has complex functions and vague concepts, making it hard to understand its uses and how to operate it.
	a12 Perceived operational complexity	Concern that operating metaverse platforms is cumbersome and time-consuming, with a high learning curve.
A6 Perceived unavailability	a13 Doubt about technological maturity	Doubt about whether current technology is mature enough to provide a smooth experience.
	a14 Device burden	Belief that additional hardware purchases or upgrades are needed, increasing financial burden.
	a15 Technology compatibility issues	Concern that existing networks or devices may not adequately support metaverse experiences, causing lag or failure.
A7 Perceived risk	a16 Data privacy risk	Fear of personal data being misused or leaked by platforms or third parties.
	a17 Identity theft risk	Worry that virtual identities could be misused, causing financial or reputational damage.
	a18 Asset security risk	Concern about virtual assets being lost, stolen, or inaccessible due to platform failure or fraud.

In this section, seven resistance factors identified among Gen Z users during the perception and anticipation stages of the metaverse were extracted through a three-stage coding process. These factors were subsequently categorized into three overarching dimensions based on their intrinsic attributes: psychological barriers (A1, A2), social value barriers (A3, A4), and cognitive barriers (A5, A6, A7). However, the specific effects of these factors on Gen Z’s resistance behaviors remain unclear, thereby necessitating further systematic investigation.

## 4 Research hypotheses and model

### 4.1 Psychological barriers

Interpersonal alienation refers to a negative emotional state arising from the estrangement between individuals and members of their social networks, including relatives, friends, neighbors, and others (Yang and Wu, 2002). In this study, interpersonal alienation specifically refers to users’ psychological perception, during the pre-use cognitive stage of the metaverse, of a lack or insufficiency in the authenticity of interpersonal interactions and the sense of emotional connection within virtual spaces. It is considered a critical psychological factor influencing users’ engagement with immersive technologies (Kim and Lee, 2020; Kye et al., 2021). Despite enhancing the sense of immersion through technologies such as VR and AR, the metaverse—characterized by highly virtualized and digitized interactions—still struggles to replicate the emotional value derived from real-world interpersonal communication (Kye et al., 2021; Kataria et al., 2023). Existing literature suggests that interpersonal alienation in intelligent digital environments can diminish users’ emotional satisfaction and may lead to distrust or indifference toward technology (Wang, 2024), subsequently triggering resistance behaviors (Wang G. et al., 2025). Slivkin et al. (2025) further highlight that some users perceive VR as a technology that fosters isolation from reality, thereby weakening their identification with it and reducing their intention to use it. Kim and Lee (2020)‘empirical findings also confirm that interpersonal alienation significantly increases consumer resistance toward self-service technologies. Accordingly, it can be inferred that when virtual social interactions in the metaverse fail to provide emotional support equivalent to that of real-world environments, members of Generation Z are more likely to experience a sense of isolation, leading to psychological resistance toward such technologies. Based on this reasoning, the following hypothesis is proposed:

*H1*: Interpersonal alienation has a significant positive effect on Generation Z’s resistance to the metaverse.

In this study, psychological burden refers to the anticipatory anxiety and mental stress arising from potential issues that may result from the use of immersive virtual technologies, such as addiction, physical discomfort, and confusion of reality. Psychological burden typically reinforces users’ dependence on existing behavioral patterns and reduces their willingness to try new technologies or services, thereby inhibiting adoption behaviors (Shin, 2010). Joo et al. (2016), using technological stress as a mediating variable, found that the psychological and physiological strain caused by technology significantly suppressed South Korean middle school teachers’ intentions to use mobile digital textbooks. In the context of the metaverse, Boccalini et al. (2024a) demonstrated that the high level of immersion in VR-based metaverse environments can indeed induce varying degrees of physical discomfort, which negatively affects users’ attitudes and intentions toward continued usage. Qin et al. (2025) further pointed out that the sense of embodiment within the metaverse may lead to identity confusion, where individuals struggle to distinguish between their physical and virtual identities, thereby intensifying psychological unease and resistance. Mvondo (2025) also identified widespread public concerns about the psychological implications of metaverse use. In particular, worries about addictive behaviors and fears of cybersickness have been confirmed as key drivers of user resistance. Therefore, it can be inferred that when members of Generation Z perceive a higher level of psychological burden, they are more likely to exhibit resistance toward adopting metaverse technologies. Based on this reasoning, the following hypothesis is proposed:

*H2*: Psychological burden has a significant positive effect on Generation Z’s resistance to the metaverse.

### 4.2 Social value barriers

Social norms refer to informal, shared understandings that govern the behaviors of members within a society—collective beliefs about which emotions, thoughts, and behaviors are considered appropriate (Turner, 1991). When an individual’s behavior deviates from established social norms, these norms—despite not being systematically analyzed by the individual—can subconsciously shape perceptions regarding the appropriateness and efficacy of the behavior in question, thus exerting a significant influence on decision-making (McDonald et al., 2013). Currently, the underlying mechanisms through which social norm conflict impacts individual behavior remain underexplored in a systematic manner. However, based on the results of the three-level coding in this study, the construct of social norm conflict can be preliminarily analyzed through two subdimensions: negative word-of-mouth and peer adoption rate.

On the one hand, prior research has shown that negative word-of-mouth (WOM) can undermine consumers’ willingness to adopt, thereby slowing the diffusion of new technologies or products (Jahanmir and Cavadas, 2018). Further, Alnoor et al. (2024) found that negative electronic word-of-mouth significantly reduces potential users’ trust in and positive perceptions of social commerce channels, which in turn suppresses their intention to use such platforms. These findings suggest that negative WOM serves as a critical inhibitory factor in technology adoption contexts. On the other hand, herd behavior theory provides a useful framework for understanding the role of peer adoption rate (Keynes, 1930). This theory posits that herd behavior is a rational response to uncertainty and limitations in individual cognition, wherein people tend to follow the actions of others, believing that others may possess more accurate or relevant information (Baddeley, 2010). A growing body of empirical research has confirmed that conformity behaviors—driven by peer influence—positively impact metaverse adoption (Al-Adwan, 2024; Balhareth et al., 2024). However, when peer adoption rates are low, the resulting lack of social information cues fails to create a compelling conformity environment, thus weakening the confidence and willingness of potential adopters. Therefore, it can be inferred that when negative word-of-mouth is prevalent and peer adoption rates are low, conflicts with prevailing social norms will more strongly inhibit Generation Z’s intention to adopt metaverse technologies. Based on this reasoning, the following hypothesis is proposed:

*H3*: Social norm conflict has a significant positive effect on Generation Z’s resistance to the metaverse.

In this study, value doubt refers to users’ doubtful or negative attitudes during the perception and anticipation stage of the metaverse, particularly regarding its practical applicability in real life, its long-term development prospects, and its respect for and preservation of existing historical and cultural connotations. Although the metaverse has been widely promoted as the technological vision of the next-generation internet, a substantial gap still remains between its envisioned applications and users’ everyday lives (Mvondo, 2025). On the one hand, some users perceive the current metaverse as being overly focused on entertainment functions and speculative hype, without demonstrating concrete capabilities to address real-world problems (Kaabachi et al., 2025). On the other hand, the long-term developmental potential and technological stability of the metaverse remain highly uncertain, further undermining users’ confidence in sustained engagement (Frank and Rudolf, 2024). Additionally, due to prevailing cultural stereotypes, some users experience a sense of cultural alienation (Al-Kfairy et al., 2025a), which further intensifies their skepticism toward the core values of the metaverse. As noted by Trieste and Turchetti (2024), value doubt can diminish the perceived credibility of information during the technology diffusion process, thereby delaying or even hindering the adoption and widespread use of emerging technologies. Similarly, empirical findings by Luong et al. (2024) identify value doubt as a critical barrier to the adoption of digital fashion technologies. Based on these insights, it is plausible to infer that when Generation Z users harbor doubts regarding the technological value of the metaverse, they are more likely to exhibit corresponding resistance behaviors. Accordingly, the following hypothesis is proposed:

*H4*: Value doubt has a significant positive effect on Generation Z’s resistance to the metaverse.

### 4.3 Cognitive barriers

In this study, perceived complexity is defined as users’ perception of the relative difficulty associated with operating the metaverse during the pre-usage cognitive stage (Rogers, 2003). Compared to simpler innovations, complex innovations are generally more difficult to adopt due to the additional requirement of acquiring new knowledge and skills (Yuen et al., 2018). Through a word cloud analysis, Rasheed et al. (2023) identified technological complexity as a critical sub-theme contributing to users’ resistance toward the adoption of artificial intelligence and robotic services. They noted that when users perceive a technology to be complicated and difficult to master, it often leads to a sense of insecurity, thereby diminishing their willingness to use it. Empirical evidence from Cham et al. (2022) further supports this view, demonstrating that in the context of mobile payment, perceived complexity exerts a significant positive effect on consumer resistance behavior. This relationship has also been validated in immersive technology settings. Abdul Waheed and Panneerselvam (2025), in an empirical study within the context of secondary education, found that teachers’ perceived complexity of VR technology significantly suppresses their adoption intention. Based on these findings, it can be inferred that in metaverse application scenarios, when members of Generation Z perceive a high level of technological complexity, their adoption behavior may also be negatively affected. Accordingly, the following hypothesis is proposed:

*H5*: Perceived complexity has a significant positive effect on Generation Z’s resistance to adopting the metaverse.

Perceived unavailability refers to users’ perception that a certain technology is difficult to use effectively due to the lack of necessary technical conditions and support (Ali et al., 2023). Ju and Lee (2021) found that the perceived unavailability of smart clothing significantly increases consumers’ resistance to innovative products. Similarly, the empirical study by Ali et al. (2023) further demonstrated that the unavailability of facilitating conditions, as a form of usage barrier, significantly inhibits users’ willingness to adopt mobile payment services. In the context of the metaverse, its immersive experience heavily relies on high-performance VR/AR devices and stable network infrastructure (Al-Kfairy et al., 2025a), which, for some users, entails additional financial costs and technical adaptation burdens (Mahmoud, 2025). Moreover, current metaverse technologies are still in a phase of continuous iteration, and widespread issues such as platform lag and interaction delays further exacerbate users’ concerns regarding usability (Hatami et al., 2024). For Generation Z users, although they generally possess high levels of digital literacy, their expectations for seamless experiences with emerging technologies are also more stringent, making them particularly sensitive to potential technological issues (Korn et al., 2024). Therefore, it can be inferred that when Generation Z users perceive a high degree of unavailability in the current metaverse, they are more likely to exhibit resistance behaviors. Based on this reasoning, the following hypothesis is proposed:

*H6*: Perceived unavailability has a significant positive effect on Generation Z’s resistance to the metaverse.

Perceived risk, as a core concept in consumer behavior research, generally refers to the uncertainty and concern perceived by consumers regarding the types and severity of potential losses prior to acquiring or using a particular product or service (Al-Kfairy et al., 2025b). A substantial body of prior research has consistently confirmed that perceived risk exerts a significant negative influence on individuals’ behavioral intentions to adopt emerging technologies (Al-Adwan, 2024). In the context of the metaverse, scholars have begun to explore the potential impact of perceived risk on users’ resistance behaviors. For instance, the empirical study conducted by Lee and Kim (2024) demonstrated that perceived risk significantly increases innovation resistance toward metaverse technologies among university students. Moreover, the study by Pillai et al. (2025) further identified perceived risk as a critical barrier preventing consumers from engaging in apparel shopping within metaverse environments. Based on the above findings, it can be inferred that perceived risk may serve as a key antecedent driving resistance behaviors toward the metaverse among Generation Z users. Based on this reasoning, the following hypothesis is proposed:

*H7:* Perceived risk has a significant positive effect on Generation Z’s resistance to the metaverse.

**Figure 1:**
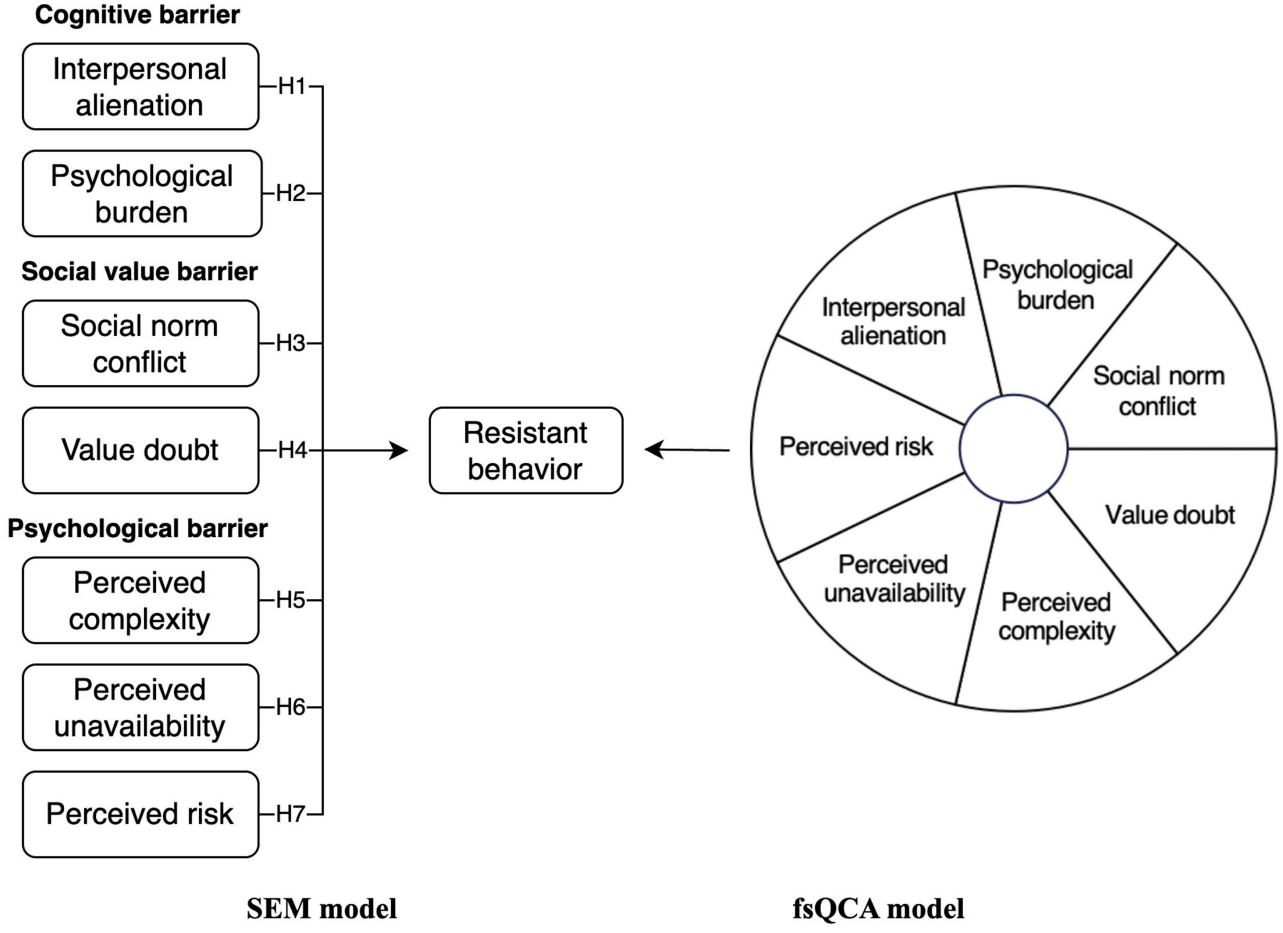
Research model.

Based on the above hypothesis, the theoretical model developed in this study is presented in Figure 1.

## 5 Materials and methods

### 5.1 Questionnaire design

The measurement scales used in this study were adapted from established and validated instruments. To ensure content validity and contextual appropriateness, the adapted questionnaire underwent multiple rounds of review and revision by six experts—two each from the fields of psychology, technology acceptance, and the metaverse. Following rigorous evaluation, the finalized instrument comprised eight constructs with a total of 26 items (see Supplementary Table S1). The revised questionnaire received unanimous approval from the experts, who affirmed its high academic applicability and research value.

### 5.2 Participants and data collection

The questionnaire employed in this study consists of four sections. The first section provides an introduction, briefly outlining the research objectives and content. The second section presented the informed consent form, which respondents were required to agree to before proceeding. The third section collected demographic information, including gender, age, educational background, income level, and occupation. The fourth section comprised items measuring the core constructs of the study, all of which were assessed using a five-point Likert scale ranging from 1 (strongly disagree) to 5 (strongly agree). To mitigate potential common method bias (CMB), anonymity and strict confidentiality were emphasized in the informed consent section, with respondents explicitly assured that they could withdraw from the survey at any time without facing any negative consequences, thereby reducing the risk of social desirability bias. Moreover, items pertaining to different constructs were randomly arranged or dispersed throughout the questionnaire to avoid response consistency caused by item clustering. In addition, all item wordings were kept neutral to minimize leading effects and reduce bias stemming from linguistic cues.

In terms of participant selection, the recruitment criteria for the questionnaire survey were consistent with those used in the prior qualitative interview phase. This stage of the study was granted ethical exemption by the Department of Global Convergence at Kangwon National University. Following the recommendations of Hair Jr et al. (2021), the minimum required sample size was calculated using G*Power software, with parameters set at f^2^ = 0.15, *α* = 0.05, and power = 0.95. The results indicated that at least 153 valid responses were necessary. Data collection was conducted from April 8 to May 10, 2025, via the Xiaohongshu platform^1^, where the research team published recruitment information and administered the survey electronically. To enhance the relevance and quality of the data, purposive sampling was employed to ensure that participants met the study’s inclusion criteria, thereby improving the validity and representativeness of the sample (Sekaran and Bougie, 2016). After the initial recruitment, the research team implemented an online verification procedure consistent with the qualitative interview stage to confirm whether respondents satisfied the requirement of never having used a metaverse platform. A total of 418 responses were collected, all from individuals who provided informed consent in electronic written form. After thorough checks for completeness and consistency, 392 valid responses were retained, meeting the sample size requirements recommended by Hair Jr et al. (2021) for subsequent data analysis.

### 5.3 Data processing and analytical methods

This study employed a mixed-method analytical approach that combined SEM with fsQCA. SEM encompasses two major methodological streams: PLS-SEM and CB-SEM (Henseler and Schuberth, 2024). Given CB-SEM’s strengths in theory testing and model fit evaluation (Vinkóczi et al., 2024), this study adopted CB-SEM as the primary method for examining net causal effects. Unlike traditional linear causal inference methods such as SEM, fsQCA is based on the principles of configurational causality, equifinality, and causal asymmetry. It allows for an inductive exploration of how multiple conditions interact to influence outcome variables (Misangyi et al., 2017). In recent years, the integration of SEM and fsQCA has gained widespread application in technology adoption and related research domains (Qu et al., 2024; Mvondo, 2025), providing robust methodological support for uncovering complex causal mechanisms. In addition, SPSS 27.0 was used for descriptive statistics and reliability testing. Confirmatory Factor Analysis (CFA) and CMB assessment were conducted using Amos 24.0 to ensure the reliability and validity of the measurement instruments and to enhance the robustness of the data.

Furthermore, prior research has indicated that individual characteristics such as age and gender may significantly influence technology resistance behaviors (Rasheed et al., 2023; Kaabachi et al., 2025). Accordingly, age and gender were included as control variables in the analysis to account for the potential confounding effects of demographic factors on the primary causal relationships.

## 6 Results

### 6.1 Respondent characteristics

Table 3 presents the demographic characteristics of the 396 valid respondents. In terms of gender, male participants accounted for a slightly higher proportion, totaling 198 individuals (50.5%). The majority of respondents were between the ages of 18 and 25, comprising 205 individuals (52.3%). Regarding occupational categories, students represented the largest group, with 116 participants (29.6%). In terms of educational background, most respondents held a bachelor’s degree, totaling 147 individuals (37.5%).

**Table 3:** Demographic characteristics of the respondents.

Items	Categories	Frequency	%
Gender	Male	198	50.5
	Female	194	49.5
Age (years)	18–25	205	52.3
	26–30	187	47.7
Occupation	Student	116	29.6
	Company employee	107	27.3
	Government employee	79	20.2
	Business owner/Management	52	13.3
	Unemployed	17	4.3
	Other	21	5.4
Education level	High school or below	32	8.2
	College diploma	128	32.7
	Bachelor’s degree	147	37.5
	Master’s degree or above	85	21.7

### 6.2 Reliability and validity test and CMB assessment

To assess the reliability of the measurement instruments, this study employed SPSS 27.0 to calculate Cronbach’s Alpha coefficients. As shown in Table 2, the Cronbach’s Alpha values for all constructs ranged from 0.826 to 0.914, exceeding the recommended threshold of 0.70 (Hair et al., 2010), indicating good internal consistency reliability of the measurement scales.

CFA was conducted using Amos 24.0 (see Table 4). The results showed that the standardized factor loadings of all measurement items exceeded the benchmark of 0.60, demonstrating strong explanatory power of the items for their respective latent constructs (Hair et al., 2010). The Composite Reliability (CR) values ranged from 0.830 to 0.918, all above the recommended threshold of 0.70, and the Average Variance Extracted (AVE) values ranged from 0.621 to 0.788, exceeding the threshold of 0.50. These results indicate good convergent validity for all constructs (Fornell and Larcker, 1981). Furthermore, as shown in Table 5, the correlations among all constructs were lower than the square roots of their respective AVEs, supporting the discriminant validity of the measurement model (Fornell and Larcker, 1981).

**Table 4:** Reliability and validity of the measurement instruments.

Constructs	Items	Mean	SD	Factor loadings	α	AVE	CR
Interpersonal alienation (IA)	IA1	3.33	0.865	0.937	0.907	0.778	0.912
	IA2	3.35	0.854	0.946			
	IA3	3.36	0.858	0.749			
Psychological burden (PB)	PB1	3.07	0.852	0.798	0.891	0.680	0.894
	PB2	2.99	0.869	0.705			
	PB3	3.05	0.817	0.855			
	PB4	3.05	0.801	0.924			
Social norm conflict (SNC)	SNC1	3.27	1.064	0.719	0.873	0.710	0.879
	SNC2	3.32	1.013	0.936			
	SNC3	3.28	1.003	0.859			
Value doubt (VD)	VD1	3.61	0.868	0.854	0.916	0.788	0.918
	VD2	3.62	0.876	0.941			
	VD3	3.63	0.932	0.866			
Perceived complexity (PC)	PC1	3.55	1.194	0.794	0.826	0.621	0.830
	PC2	3.55	1.170	0.708			
	PC3	3.62	1.110	0.855			
Perceived unavailability (PUN)	PUN1	3.40	0.908	0.748	0.855	0.670	0.859
	PUN2	3.41	0.863	0.861			
	PUN3	3.41	0.877	0.843			
Perceived risk (PR)	PR1	3.35	0.839	0.937	0.907	0.768	0.908
	PR2	3.27	0.882	0.826			
	PR3	3.35	0.849	0.863			
Resistance behavior (RB)	RB1	3.53	0.964	0.923	0.914	0.731	0.916
	RB2	3.54	1.006	0.821			
	RB3	3.56	0.961	0.834			
	RB4	3.53	1.001	0.839			

SD: Standard deviation, α: Cronbach’s alpha.

**Table 5:** Discriminant validity test.

	RB	PR	PUN	PC	VD	SNC	PB	IA
RB	**0.855**							
PR	0.317	**0.876**						
PUN	0.323	0.132	**0.819**					
PC	0.220	0.151	0.091	**0.788**				
VD	0.310	0.121	0.180	0.027	**0.888**			
SNC	0.326	0.105	0.136	0.124	0.100	**0.843**		
PB	0.382	0.153	0.123	0.191	0.154	0.165	**0.825**	
IA	0.352	0.136	0.191	0.139	0.078	0.209	0.108	**0.882**

Bold values represent the square root of the AVE.

Following the evaluation criteria adopted by Qu et al. (2024), Hu et al. (2025), and Çelik and Ayaz (2025), the overall model fit indices of the measurement model fall within acceptable ranges (see Table 6), further confirming the adequacy and robustness of the measurement structure.

**Table 6:** CFA model fit, ULMF test, and SEM model fit.

Fit index	Recommended value	CFA model (8-factor)	ULMF test (8-factor + Method factor)	SEM model
χ^2^/df	<3	1.233	1.176	1.459
GFI	>0.9	0.940	0.947	0.914
RMSEA	<0.08	0.024	0.021	0.034
IFI	>0.9	0.990	0.993	0.976
CFI	>0.9	0.990	0.993	0.976
TLI	>0.9	0.988	0.991	0.973

Since all data in this study were obtained from respondents’ self-reports, the potential influence of common method bias cannot be fully ruled out, despite the various control measures adopted during the questionnaire design stage (Podsakoff et al., 2003). To mitigate this risk, a common method factor was introduced into the eight-factor CFA model by loading all measurement items onto their respective latent constructs as well as onto the common method factor (Lindell and Whitney, 2001), thereby enabling an unmeasured latent method factor (ULMF) test (Richardson et al., 2009). The results indicate that, after including the common method factor, the overall model fit indices (see Table 6) did not differ significantly from those of the original model. This finding suggests that common method bias is not a serious concern in the present study (Richardson et al., 2009).

### 6.3 SEM analysis results

**Figure 2:**
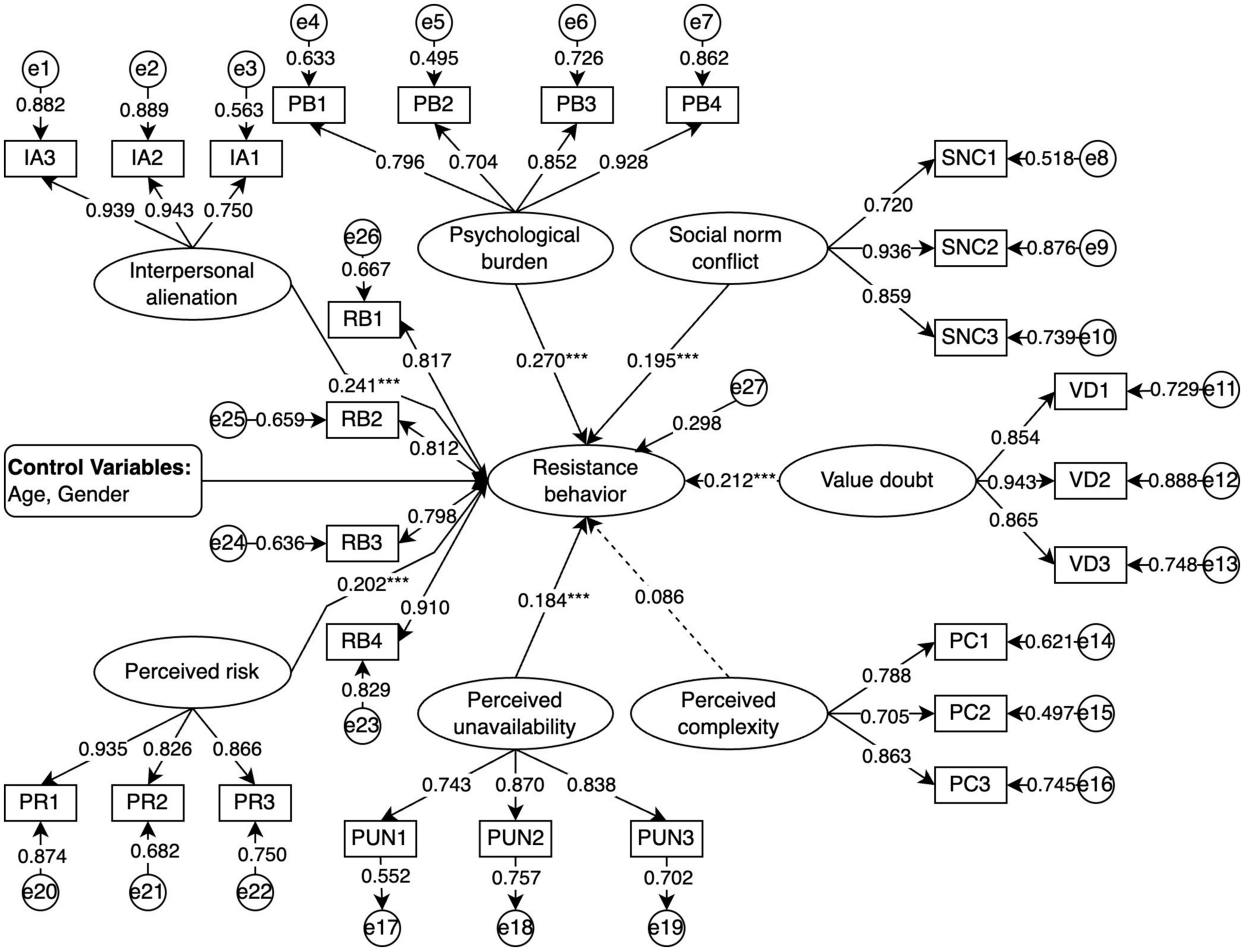
Results of model analysis (***p < 0.001, the dashed line indicates that the hypothesis is not supported).

The fit indices of the SEM indicate a good model fit (Table 6). The results of the SEM path significance tests (Table 7; Figure 2) show that IA (*β* = 0.241, *p* < 0.001), PB (*β* = 0.270, *p* < 0.001), SNC (*β* = 0.195, *p* < 0.001), VD (*β* = 0.212, *p* < 0.001), PUN (*β* = 0.184, *p* < 0.001), and PR (*β* = 0.202, *p* < 0.001) exert significant negative effects on RB. In contrast, the effect of PC on RB (*β* = 0.086, *p* = 0.096) is not statistically significant. Therefore, hypotheses H1–H4, H6, and H7 are supported, whereas H5 is not supported. Additionally, the analysis of control variables indicates that neither age nor gender has a significant impact on RB.

**Table 7:** Results of hypothesis testing.

Hypothesis	Path	B	*β*	C. R.	*p*	Results
H1	IA → RB	0.252	0.241	4.806	***	Supported
H2	PB → RB	0.337	0.270	5.272	***	Supported
H3	SNC → RB	0.216	0.195	3.847	***	Supported
H4	VD → RB	0.243	0.212	4.300	***	Supported
H5	PC → RB	0.077	0.086	1.665	0.096	Rejected
H6	PUN → RB	0.231	0.184	3.559	***	Supported
H7	PR → RB	0.219	0.202	4.085	***	Supported

B: Unstandardized coefficient, *β*: Standardized coefficient. C. R.: Critical ratio, ***p < 0.001.

### 6.4 fsQCA results

Given the limitations of SEM in capturing nonlinear relationships among independent variables (Qu et al., 2024), this study further adopts fsQCA to complement the evaluation of the configurational effects of the predictors.

#### 6.4.1 Data calibration

Prior to conducting fsQCA, the original data obtained from the five-point Likert scale were transformed into fuzzy set membership scores ranging from 0 to 1 (Zhao et al., 2024). First, the average scores of each latent construct’s dimensions were computed to serve as representative indicators of the respective variables (Zhao et al., 2024). Second, based on Ragin (2008)‘s three anchor points—5% (full non-membership), 50% (crossover point), and 95% (full membership)—the data were calibrated using the Calibrate function in the fsQCA 3.0 software. Finally, to ensure the robustness of the analysis and to avoid case exclusion due to exact crossover values, a constant of 0.001 was added to all fuzzy scores that were exactly 0.5 (Fiss, 2011). The detailed calibration results are presented in Table 8.

**Table 8:** Data calibration.

Before calibration	Fuzzy-set calibration			After calibration	Descriptive statistics			
	FM	CP	FNM		Mean	SD	Min	Max
IA	5	3	2	FIA	0.579	0.253	0	0.95
PB	4	3	2	FPB	0.526	0.327	0	1
SNC	5	3.333	1.667	FSNC	0.486	0.292	0.01	0.95
VD	5	4	2	FVD	0.419	0.270	0.01	0.95
PC	5	3.667	1.667	FPC	0.522	0.304	0.02	0.05
PUN	5	3.333	2	FPUN	0.506	0.276	0.01	0.95
PR	5	3	2	FPR	0.572	0.263	0	0.95
RB	4.75	3.875	2	FRB	0.468	0.295	0.01	0.98

FM: Full membership, CP: Crossover point, FNM: Full non-membership, SD: Standard deviation.

#### 6.4.2 Necessary condition analysis

The purpose of necessary condition analysis is to identify which predictor variables are indispensable drivers for achieving a high level of resistance behavior. When the consistency and coverage of a predictor variable exceed 0.9, the variable can be regarded as a “necessary condition” (Dul, 2016). The results of the necessary condition analysis (Table 9) indicate that achieving a high level of resistance behavior does not depend on any single predictor variable as a necessary condition.

**Table 9:** Results of necessary condition analysis.

Conditions	Consistency	Coverage
FIA	0.829	0.670
~FIA	0.558	0.619
FPB	0.758	0.674
~FPB	0.548	0.541
FSNC	0.732	0.705
~FSNC	0.634	0.576
FVD	0.670	0.748
~FVD	0.704	0.567
FPC	0.758	0.679
~FPC	0.585	0.572
FPUN	0.766	0.708
~FPUN	0.605	0.573
FPR	0.812	0.663
~FPR	0.558	0.610

#### 6.4.3 Sufficient condition analysis

Following the procedures outlined by Ragin (2008), this study employed the fsQCA 3.0 software to construct a truth table comprising 2^k^ rows (where “k” represents the number of predictor variables). Each row corresponds to a unique configuration of the seven predictor variables and includes the frequency of cases that exhibit this configuration as well as a consistency score. Given the sample size exceeds 150 cases, and in line with the recommendations of Fiss (2011) and Pappas and Woodside (2021), the frequency threshold was set at 3 and the consistency threshold at 0.9, in order to eliminate low-quality configurations. The analysis generated three types of solutions: the complex solution, the intermediate solution, and the parsimonious solution. Among these, the intermediate solution was selected as the main interpretative basis of the study, as it retains a high level of explanatory power while better revealing the key causal mechanisms (Ragin, 2008). Furthermore, by conducting counterfactual comparisons between the intermediate and the corresponding parsimonious solutions, core and peripheral conditions within each configuration were identified (Fiss, 2011). It is important to note that in some instances, a single intermediate solution corresponded to multiple parsimonious solutions. Following the technical guidelines proposed by Pappas and Woodside (2021), all conditions appearing in the set of parsimonious solutions were treated as core conditions in this study.

Table 10 presents nine causal configurations that lead to high levels of resistance behavior formation. According to the criteria proposed by Dul (2016), a configuration is considered to have strong explanatory power if its consistency is not lower than 0.8 and its coverage is not lower than 0.2. The results indicate that all identified causal paths meet these criteria. Specifically, the overall solution has a consistency of 0.825 and a coverage of 0.682, suggesting that the nine configurations collectively possess strong explanatory power and robustness. Among all the paths, configuration S3 exhibits the highest raw coverage (0.450), accounting for the largest number of cases, with a consistency of 0.874, making it the optimal explanatory path. In addition, configurations S1, S2, S4, S8, and S9 all demonstrate raw coverage exceeding 0.40 (ranging from 0.404 to 0.446) and consistency between 0.872 and 0.901, further confirming their strong explanatory capacity. In contrast, configurations S5 through S7 show slightly lower raw coverage values (ranging from 0.306 to 0.374), but maintain high consistency levels (between 0.872 and 0.910). Notably, configuration S6 achieves the highest consistency among all paths, indicating a particularly robust causal relationship. In terms of the frequency of core conditions, perceived complexity, psychological burden, and value doubt appear as core conditions in more than half of the configurations, underscoring their critical role in the formation of high levels of resistance behavior. It is also worth noting that configuration S5 contains a negated condition, suggesting that even under low levels of perceived complexity, strong resistance behavior can still emerge if other key factors are sufficiently strong.

**Table 10:** Configurations of conditions.

Conditions	Solutions								
	S1	S2	S3	S4	S5	S6	S7	S8	S9
FIA	▲	▲	▲			▲	▲	▲	▲
FPB	●	●			●	●		●	●
FSNC				▲		●	▲	●	
FVD	●	●	●	●	●	●	●		
FPC	●		●	●	〇		●		●
FPUN					▲	●	▲	●	●
FPR		▲	▲	▲	▲			▲	▲
Consistency	0.894	0.888	0.874	0.872	0.897	0.910	0.904	0.901	0.895
Raw coverage	0.422	0.446	0.450	0.405	0.306	0.374	0.370	0.404	0.419
Unique coverage	0.009	0.012	0.018	0.013	0.009	0.011	0.006	0.009	0.019
Solution consistency	0.825								
Solution coverage	0.682								

● indicates the presence of a core condition, ▲ represents the presence of a peripheral condition, 〇 indicates the absence of a core condition, and a blank space implies the condition is irrelevant.

## 7 Discussion

This study employed a mixed-methods approach to systematically investigate Generation Z’s resistance behavior toward the metaverse and its underlying mechanisms. In the qualitative phase, based on Generation Z respondents’ perceptions and anticipations of the early forms of the metaverse, seven key perceptual factors were identified through three-stage coding based on grounded theory: interpersonal alienation, psychological burden, social norm conflict, value skepticism, perceived complexity, perceived unavailability, and perceived risk. In the quantitative phase, results from SEM revealed several important insights. First, interpersonal alienation significantly increases Generation Z’s resistance to the metaverse, supporting the perspectives of Kim and Lee (2020) and Slivkin et al. (2025). This indicates that within metaverse usage anticipations, virtual environments fail to provide emotional satisfaction comparable to real-world social interactions, thereby weakening Generation Z’s adoption motivation. Second, the positive influence of psychological burden was also significant, corroborating findings by Mvondo (2025) and Qin et al. (2025). Notably, despite their lack of actual usage experience, Generation Z still exhibited considerable psychological burden, suggesting that their concerns stem primarily from social discourse and self-expectations rather than real usage experience. Social norm conflict also had a significant impact on resistance behavior, aligning with the conclusions of McDonald et al. (2013). The significance of value skepticism is supported by Trieste and Turchetti (2024) and Luong et al. (2024). Within the Chinese cultural context, the influence of social norm conflict and value skepticism may be further amplified. Under collectivist orientations, individuals rely more heavily on group opinions and peer adoption rates (Fan et al., 2018). Consequently, negative word-of-mouth and low penetration rates of the metaverse exert stronger effects on Chinese Generation Z’s resistance behaviors. Moreover, having grown up at the intersection of traditional and digital cultures (Zhang et al., 2025), Chinese Generation Z are more sensitive to cultural simplification or distortion in virtual environments, thereby deepening their value doubt. In addition, perceived unavailability was likewise significant, echoing findings from Ju and Lee (2021) and Ali et al. (2023), which indicate that technological barriers, device burdens, and platform instability constitute important practical obstacles shaping resistance behaviors during the anticipation stage. Perceived risk also significantly influenced resistance behavior, in line with research by Lee and Kim (2024) and Pillai et al. (2025). This demonstrates that concerns related to identity, privacy, and asset security inhibit Generation Z’s adoption intention during their anticipation stage of the metaverse. However, Jaradat et al. (2018) argue that in certain contexts, if the perceived benefits are substantial or the risks are manageable, perceived risk may instead act as a stimulus for positive evaluation. Hence, the influence of risk may be context-dependent. In contrast, perceived complexity did not exhibit a significant effect on resistance behavior. This result is consistent with the findings of Cham et al. (2024). Nevertheless, divergent views exist. For example, Cham et al. (2022), in a study on mobile payment contexts, found that perceived complexity significantly increased user resistance, emphasizing that in scenarios with high operational thresholds or unclear user expectations, complexity can serve as a major adoption barrier. More controversially, Himanshu et al. (2025), in a metaverse usage context similar to that of the current study, reported an opposite finding: perceived complexity had a positive effect on behavioral intention. This divergence may be attributed to the fact that their research sample comprised individuals with prior experience using the metaverse. For this group, complexity may instead serve as a positive cue reflecting system sophistication and value density, thereby enhancing their motivation to engage with the technology. These contrasting results indicate that the role of perceived complexity is not fixed; rather, its effects may vary depending on sample characteristics and research contexts.

The fsQCA configuration analysis revealed nine equifinal pathways leading to resistance behavior, which effectively complement the findings from the SEM analysis. Overall, the conditions of interpersonal alienation, psychological burden, social norm conflict, value skepticism, perceived unavailability, and perceived risk recurrently appeared across four to seven configurations, underscoring their central role in predicting resistance behavior—consistent with the significant effects of these variables identified in the SEM results. Among them, psychological burden and value skepticism emerged as core conditions in most pathways and were also key predictors in the SEM model, further reinforcing their theoretical explanatory power. However, notable differences also emerged between the fsQCA and SEM findings. First, pathway S5 revealed a reverse condition: even when individuals perceived low complexity, they could still exhibit strong resistance behaviors if psychological burden, value skepticism, perceived unavailability, and perceived risk were simultaneously high. Second, while interpersonal alienation was identified as the strongest predictor of resistance behavior in the SEM model, it was treated as a peripheral condition in most fsQCA pathways—a pattern similarly observed for perceived risk. Finally, although perceived complexity had no significant impact in the SEM analysis, it appeared as a core condition in six fsQCA configurations. This suggests that perceived complexity may function as a prerequisite or synergistic factor within more intricate causal structures. Such non-linear interaction effects highlight the limitations of SEM in capturing complex causal mechanisms (Qu et al., 2024).

### 7.1 Theoretical implications

This study offers several key theoretical contributions, articulated in the following three aspects. First, it enriches the theoretical landscape of user behavior research in the metaverse domain. Although scholarly interest in the metaverse has surged in recent years, mainstream studies have predominantly focused on technological evolution (Wang Y. et al., 2025), business models (Qadir and Fatah, 2023), and usage motivations (Wu and Yu, 2024), while largely neglecting the “negative behavior” dimension of user resistance. In contrast, this study centers on Generation Z—an essential user cohort that commands considerable theoretical and practical attention (Boccalini et al., 2024b)—and investigates the question of why they choose not to engage with metaverse platforms. By systematically identifying perceptual barriers that trigger resistance behaviors during the anticipation stage, it extends the theoretical boundaries of metaverse user behavior research and establishes a solid conceptual foundation for subsequent cross-platform comparative studies on resistance mechanisms.

Second, this study advances the theoretical development of resistance behavior research. Existing studies on resistance behavior often derive variables within the IRT framework (Mvondo, 2025; Kaabachi et al., 2025), typically limiting their analyses to functional or psychological dimensions, while overlooking the integrated effects of emotional identification, sociocultural norms, and cognitive load. By employing open-ended interviews and coding techniques within grounded theory, this study inductively identifies key resistance constructs from the authentic discourses of Generation Z users. This approach not only partially responds to the theoretical propositions of IRT but also compensates for its limitations in variable identification and contextual interpretation. Specifically, the “psychological barriers” identified in this study echo the IRT framework but further evolve into context-specific categories under the metaverse setting, namely “interpersonal alienation” and “psychological burden.” At the same time, functional barriers posited by IRT did not emerge as significant resistance factors among tech-savvy Generation Z users. Instead, the study proposes “social value barriers” and “cognitive barriers” as more contextually relevant constructs, thereby effectively extending the IRT framework and providing novel explanatory perspectives on the distinctive response mechanisms of Generation Z in emerging technology environments.

Third, this study achieves a methodological integration of qualitative and quantitative approaches, as well as symmetric and asymmetric analytical pathways, thereby enhancing the theoretical explanatory power of complex causal mechanisms. Specifically, based on the identification of key variables through qualitative research, the study combines SEM with fsQCA to uncover both the configurational pathways and substitutive logics among various resistance factors. This dual-method approach strengthens the theoretical robustness and captures the practical complexity of the findings, directly addressing the research concern of “multiple configurational causality” in technology acceptance studies (Qu et al., 2024; Zhao et al., 2024). As such, it offers a methodological innovation for future research on user behavior mechanisms in the metaverse context.

### 7.2 Practical implications

This study offers several practical implications, particularly in the following three areas. First, it provides empirical support for the formulation of differentiated operational strategies for metaverse platforms. The study identifies six distinct factors—interpersonal alienation, psychological burden, social norm conflict, value skepticism, perceived unavailability, and perceived risk—that independently shape resistance behaviors among Generation Z users during the anticipation stage. Accordingly, in both product design and service promotion, metaverse platforms should adopt a user-centered approach that addresses Generation Z’s subjective experiences. Specifically, targeted strategies should be implemented to alleviate psychological pressure, enhance authentic social interaction, reconstruct cultural identity, optimize security mechanisms, and improve technological stability and device compatibility. Furthermore, the findings reveal the presence of multiple concurrent and substitutable causal pathways, as well as nonlinear interactions among various resistance factors. This indicates that relying solely on the improvement of individual platform features is unlikely to significantly mitigate resistance behaviors. Developers should therefore pursue differentiated designs and customization tailored to varying user characteristics and barrier configurations (Zhao et al., 2024). Building upon the configurational pathways uncovered in this study, platforms are advised to advance collaboratively across multiple dimensions such as technological usability, alignment with social norms, cultural meaning construction, and psychological support mechanisms to establish a multi-level, cross-modal user value system that more precisely resonates with users’ cognitive traits and behavioral preferences.

Second, given the currently low adoption rate of metaverse platforms among Generation Z users (Korn et al., 2024; Kaabachi et al., 2025), this study provides strategic insights for user retention and reactivation within this demographic. Unlike the traditional growth logic that emphasizes technological advancement as the primary driver (Wang Y. et al., 2025), the findings suggest that, in contexts where actual usage experience is lacking, Generation Z tends to be more influenced by social-cognitive cues and affective evaluation mechanisms when engaging with metaverse technologies. Therefore, in platform communication and community management targeting Generation Z, greater emphasis should be placed on the cultivation of positive word-of-mouth, the creation of an inclusive and diverse community atmosphere, and the design of content with strong real-world relevance and cultural resonance. These approaches can enhance Generation Z’s sense of value alignment with the platform. For users who have already exhibited resistance tendencies, personalized intervention strategies are recommended. For example, offering gradual immersive experiences and simplifying operational processes can help reduce psychological barriers and alleviate technology-related anxiety. Such tailored measures may increase the willingness of resistant users to reengage and facilitate their pathway back to active platform participation.

Finally, this study offers practical insights for policy formulation and public communication practices. As the core demographic of the future digital society (Yani and Santosa, 2024), Generation Z’s acceptance of metaverse technologies not only shapes the developmental trajectory of related platforms but also has far-reaching implications for the evolution of technology governance structures and digital education policies. Policymakers should adopt a multifaceted approach—encompassing psychological adaptation, cultural guidance, and social dialogue—to develop more tailored mechanisms for technology diffusion that align with the characteristics of Generation Z. For instance, mainstream media and educational platforms can be leveraged to strengthen the perceived real-world relevance of the metaverse, promote cultural diversity, and reinforce ethical norms surrounding its use. Such efforts can enhance public meaning-making and foster value alignment with emerging technologies. In parallel, the development of a collaborative governance framework should be promoted, encouraging joint participation from enterprises, educational institutions, and regulatory bodies in shaping public discourse around digital environments. This inclusive approach can facilitate the construction of a more resilient social negotiation mechanism that bridges technological advancement with user adaptation, ensuring more sustainable and equitable digital transformation.

### 7.3 Limitations and directions for future research

This study still has several limitations that warrant further improvement in future research. First, the sample in this study was primarily drawn from mainland China. Given China’s unique context in terms of digital culture and media regulation, factors such as social norm conflicts and value questioning may be more salient under a collectivist cultural setting. Therefore, the findings may exhibit cultural specificity. Future studies could further test and compare the conclusions in cross-cultural contexts to enhance their generalizability and external validity. Second, the SEM results indicate that perceived complexity did not exert a significant effect on resistance intention. This finding may be attributable to the generally high level of technological literacy among Generation Z, which diminishes the inhibitory role of perceived complexity in behavioral decision-making. Alternatively, it may stem from the participants’ lack of actual usage experience, rendering their evaluations of complexity largely conceptual. Future research could further explore differential effects across groups with varying levels of technological literacy or actual experience, thereby enriching the interpretation of this result. Third, although this study employed a mixed-methods approach to strengthen the comprehensiveness of causal and configurational analyses, the conclusion that the “independent effect of perceived complexity is insignificant” remains limited when drawn solely from SEM results. Future research may incorporate multimodal techniques such as eye-tracking and physiological feedback to capture user behavioral dynamics and emotional fluctuations during interaction processes in a more multidimensional manner. Such approaches would deepen the understanding of the mechanisms through which perceived complexity influences user behavior and enhance the processual insights of the overall research conclusions. Finally, this study only controlled for age and gender. However, factors such as individual innovativeness, prior technology usage experience, and personality traits may also play a crucial role in shaping users’ resistance behaviors. Future studies could integrate a broader set of control and moderating variables to further improve the explanatory power of the model and strengthen the robustness of the conclusions.

## Data Availability

The datasets presented in this study can be found in online repositories. The names of the repository/repositories and accession number(s) can be found below: Figshare, http://doi.org/10.6084/m9.figshare.29557772.

## Footnotes

^1^ https://www.xiaohongshu.com
