# Supplementary material for: Metaverse? No, thanks! Exploring the mechanisms behind Generation Z’s resistance behavior
Source: Front Psychol. 2025 Nov 3;16:1672330. doi: 10.3389/fpsyg.2025.1672330 (PMC12620464; doi:10.3389/fpsyg.2025.1672330)
Supplement: Supplementary file 1 [file Table_1.docx]

Supplementary Material

# Supplementary Table

**Supplementary Table S1. Measurement items.**

| **Constructs** | | **Measurement items** | **Sources** |
| --- | --- | --- | --- |
| Interpersonal alienation  (IA) | IA1 | I believe that the sense of social companionship in the metaverse is not as genuine as in real life. | Slivkin et al. (2025) |
|  | IA2 | I feel that using the metaverse would gradually distance me from people in the real world. |  |
|  | IA3 | I'm worried that being immersed in the metaverse might lead to social exclusion in real life. |  |
| Psychological burden  (PB) | PB1 | I'm concerned that I might become dependent on the metaverse. | Mvondo (2025)  Kaabachi et al. (2025) |
|  | PB2 | I'm afraid I might spend too much time in the metaverse. |  |
|  | PB3 | I'm afraid of experiencing physical discomfort while using the metaverse. |  |
|  | PB4 | I'm concerned that using the metaverse may blur the boundaries between my virtual and real identities. |  |
| Social norm conflict  (SNC) | SNC1 | There is a large amount of negative media coverage about the metaverse. | Al-Adwan (2024)  Kaabachi et al. (2025) |
|  | SNC2 | The metaverse has a relatively poor public reputation. |  |
|  | SNC3 | Very few of my friends use the metaverse, so I'm not inclined to use it either. |  |
| Value doubt  (VD) | VD1 | I am skeptical about the practicality of the metaverse and doubt its ability to effectively solve real-world problems. | Kaabachi et al. (2025)  Nie et al. (2025) |
|  | VD2 | I question whether the metaverse is sustainable and has long-term value. |  |
|  | VD3 | I think cultural representation in the metaverse lacks diversity. |  |
| Perceived complexity (PC) | PC1 | I find the metaverse relatively complex and difficult to use. | Pillai et al. (2025) |
|  | PC2 | I find it difficult to understand the functions and operating procedures of the metaverse. |  |
|  | PC3 | I'm worried that I might encounter errors while using the metaverse and struggle to resolve them promptly. |  |
| Perceived unavailability  (PUN) | PUN1 | I doubt whether the metaverse is technically mature and practically feasible. | Mvondo (2025)  Sowmya et al. (2024)  Kaabachi et al. (2025) |
|  | PUN2 | There are some financial barriers (such as the cost of VR/AR headsets and subscription fees) that limit my ability to use the metaverse. |  |
|  | PUN3 | I'm concerned about not having adequate internet and device conditions to access the metaverse. |  |
| Perceived risk  (PR) | PR1 | I'm worried about the authenticity of others' identities in the metaverse and the potential risks this may bring. | Nie et al. (2025) |
|  | PR2 | I'm concerned that my personal information might be leaked while using metaverse products. |  |
|  | PR3 | I'm worried about the security of virtual assets within metaverse products. |  |
| Resistance behavior  (RB) | RB1 | I am very likely to refuse to use the metaverse. | Mvondo (2025)  Kaabachi et al. (2025) |
|  | RB2 | The metaverse is not suitable for me. |  |
|  | RB3 | I have no plans to use the metaverse in the near future. |  |
|  | RB4 | I am very likely to oppose statements that exaggerate the benefits of the metaverse. |  |
